# Effects of low-density urbanization on genetic structure in the Song Sparrow

**DOI:** 10.1371/journal.pone.0234008

**Published:** 2020-06-12

**Authors:** Valerie N. Brewer, Samuel J. Lane, Kendra B. Sewall, Karen E. Mabry

**Affiliations:** 1 Department of Biology, New Mexico State University, Las Cruces, NM, United States of America; 2 Department of Biological Sciences, Virginia Polytechnic Institute and State University, Blacksburg, VA, United States of America; Bowling Green State University, UNITED STATES

## Abstract

Urbanization fragments landscapes and can impede the movement of organisms through their environment, which can decrease population connectivity. Reduction in connectivity influences gene flow and allele frequencies, and can lead to a reduction in genetic diversity and the fixation of certain alleles, with potential negative effects for populations. Previous studies have detected effects of urbanization on genetic diversity and structure in terrestrial animals living in landscapes that vary in their degree of urbanization, even over very short distances. We investigated the effects of low-intensity urbanization on genetic diversity and genetic structure in Song Sparrows (*Melospiza melodia*). We captured 208 Song Sparrows at seven sites along a gradient of urbanization in and around Blacksburg, VA, USA, then genotyped them using a panel of fifteen polymorphic microsatellite loci. We found that genetic diversity was comparable among the seven study sites, and there was no evidence of genetic structuring among sites. These findings suggest that over a gradient of urbanization characterized by low density urban development, Song Sparrows likely exist in a single panmictic population.

## Introduction

Urbanization and habitat fragmentation are major concerns in a changing world, and can have large effects on population genetic diversity and structure. Habitat loss and fragmentation are characterized by the disruption of a previously continuous landscape and the separation of natural areas into smaller patches with reduced area and increased isolation [1]. Human need for more land and resources has led to habitat fragmentation through expanded development into previously rural areas, known as urbanization. Anthropogenic factors such as highways, bridges, and gaps in natural cover may impede the movements of individuals through the environment; even highly-mobile organisms such as birds may have their movements restricted by anthropogenic habitat changes [2–4], which can promote genetic isolation due to restricted interactions between individuals. Reduced connectivity may lessen the genetic diversity of members of a breeding population, impact gene flow and allele frequencies, and can lead to allele fixation, with potential negative consequences for animal populations [5–7].

Habitat changes due to urbanization have been shown to affect genetic diversity and structure in a variety of animal species. For example, an evaluation of genetic structure and diversity in several species of small terrestrial vertebrates in Los Angeles, CA suggested that high-density urbanization may have a severe effect on animal populations, resulting in reduced genetic diversity and increased genetic structure in more isolated patches [8]. Other recent studies have shown dramatic effects of urbanization on population genetic structure in small terrestrial animals living in highly urbanized environments [9,10]. However, the effects of urbanization on genetic diversity and genetic structure are not limited to species with limited vagility; such effects have also been observed in a range of bird species. For example, Song Sparrows in habitats across a gradient of urbanization characterized by intensive anthropogenic development (Seattle, WA) had levels of genetic differentiation among sites that were correlated with the age of urban development such that populations in urban areas that had been developed for a longer period of time had higher levels of genetic structure (although genetic diversity was comparable across sites) [11]. Similarly, Great Tits (*Parus major*) surveyed across an urbanization gradient displayed a weak but detectable effect of urbanization on genetic structure and a small reduction in genome-wide diversity in more urban areas [12]. Similarly, House Sparrows (*Passer domesticus*) across a gradient of urbanization exhibited greater genetic structure in the urban habitat center, yet there was no significant effect on genetic diversity [13,14]. The range of results seen in these and similar studies indicates that factors such as the life history of the species and the intensity, duration, and magnitude of anthropogenic change can play a role in the impact of urbanization on population genetics[15].

Fully understanding the consequences of urbanization for wildlife genetic diversity requires examining population genetic structure in species living along gradients that vary in urbanization intensity [15], as well as investigating this question in species that are widely-distributed, seemingly tolerant to disturbance, or even considered pests [16–18]. To contribute to this research effort, we investigated the population genetic structure of Song Sparrows across a gradient of low intensity urbanization [19]. Song Sparrows are small songbirds that are abundant throughout North America [20]. In Blacksburg, Virginia, USA, where our study was conducted, Song Sparrows are year-round residents (KB Sewall personal observation). Song Sparrows are territorial and highly philopatric [21]. Further, the relatively short natal dispersal distances observed in the species [21,22] paired with the lack of migration by Song Sparrows in the region of this study (KB Sewall personal observation) and previous detection of fine-scale genetic structure in Song Sparrows at distances of 2–4 km [23,24] makes them a valuable system in which to examine the genetic implications of low-density urbanization for mobile species. Birds in our study area show marked behavioral differentiation between urban and rural sites: urban male Song Sparrows are significantly more territorially aggressive than are rural males [25], and urban males are more bold, provide more parental care, and have larger territories than do male birds in rural habitats (KB Sewall personal observation). The observed behavioral differences between urban and rural birds may be due to plastic responses to environmental variation, genetic variation, or a combination of influences. Further, the presence of habitat-related behavioral differences between nearby sites raises the possibility that even across short distances, genetic differentiation may be present. We used microsatellite genotypes to compare the genetic diversity and population genetic structure of 208 birds over approximately 15 km, surveying birds living in undisturbed riparian areas, farm land, and suburban habitats that we describe here as rural, intermediate, and suburban, respectively. If low-intensity urbanization affects genetic diversity and genetic structure in this population, we would expect to see lower genetic diversity and increased levels of genetic differentiation in birds sampled at sites with higher levels of urbanization.

## Materials and methods

### Study site and landscape categorization

**Fig 1:**
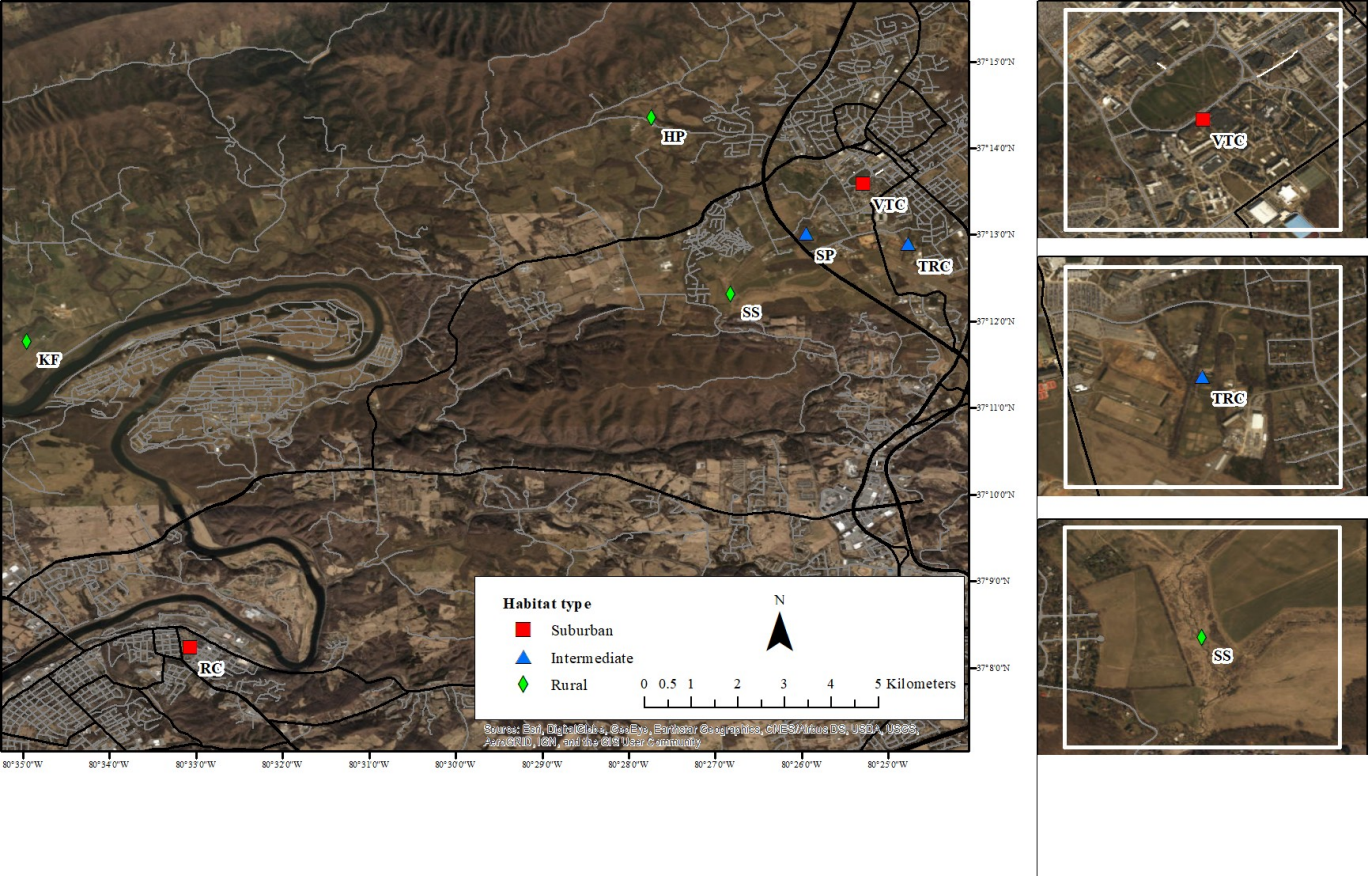
Map of study sites. Satellite image of the study sites with a marker placed in the approximate center of each labeled study site. Site names on the map are abbreviated as follows: Radford Campus (RC), Virginia Tech Campus (VTC), Turfgrass Research Center (TRC), Smithfield Plantation (SP), Heritage Park (HP), Kentland Farm (KF), and Stream Site (SS). Imagery base map source: Esri, DigitalGlobe, GeoEye, Earthstar Geographics, CNES/Airbus DS, i-cubed, USDA FSA, USGS, AEX, Getmapping, Aerogrid, IGN, IGP, swisstopo, and the GIS User Community. Street layer source: Esri and TomTom.

We conducted our research at seven sites across an urbanization gradient in and around Blacksburg, VA: two suburban, two intermediate, and three rural (Fig 1 and Table 1). These sites were originally selected for a set of previous studies, which detected significant effects of urbanization on aggressive behavior by territorial males [25] and sought to identify the physiological mechanisms underlying those differences [19]. The “suburban” and “rural” sites were chosen to represent the extremes of local values of impervious surface, with “intermediate” sites having a proportion of impervious surface between those extremes; final site selection was also influenced by accessibility and landowner permissions. To quantify land use at each site, we used the semi-automated method described by Seress et al. [26]. Estimates of land use by this method are strongly correlated with results from finer-resolution geoinformatic measurements [26]. Briefly, this approach divides an aerial image of a 1 km^2^ area around each site into 100 m × 100 m cells and scores the abundance of vegetation, buildings, and paved surfaces, such as roads and parking lots, in each cell. These scores are used to calculate an ‘urbanization index’ for each site using principal component analysis (PCA; Table 1) [19,25]. The city of Radford, VA, the location of Radford Campus, was founded in 1887 and had a density of 641.7 people per km^2^ in 2010 and Blacksburg, the location of Virginia Tech Campus, was founded in 1798 and had a density of 827.2 people per km^2^ in 2010 [27].

**Table 1:** Location, sample size, urbanization index, mean observed (*H*_o_) and expected (*H*_e_) heterozygosity across 13 microsatellite loci, fixation index (*F*_IS_), mean number of alleles/locus (Na) for each site.

Site name	Latitude	Longitude	Habitat type	Sample Size	Urbanization index	*H*_o_	*H*_e_	*F*_IS_	*N*_a_
Radford Campus	37.1386° N	80.5507° W	Suburban	20	3.35	0.62	0.69	0.09	7.15
Virginia Tech Campus	37.2276° N	80.4220° W	Suburban	44	3.05	0.66	0.72	0.06	9.70
Turfgrass Research Center	37.2148° N	80.4125° W	Intermediate	18	0.07	0.58	0.69	0.18	7.39
Smithfield Plantation	37.2177° N	80.4326° W	Intermediate	20	-0.65	0.63	0.72	0.16	8.62
Heritage Park	37.2441° N	80.4594° W	Rural	23	-1.73	0.67	0.69	0.03	7.69
Kentland Farm	37.2009° N	80.5774° W	Rural	39	-2.03	0.66	0.72	0.08	9.92
Stream Site	37.2083° N	80.4467° W	Rural	44	-2.20	0.65	0.72	0.10	9.77

The urbanization indices are taken from Davies and Sewall [21].

### Sample collection

Genetic samples of Song Sparrows were obtained from blood collected during the 2014–2017 breeding seasons (March–June each year). Birds were captured primarily through mist netting accompanied by song playback; this method, which relies on territoriality, primarily attracts males. Mist nets were set up at the center of each study site; we did not locate the nests of individual birds. After capture, approximately 150 μL of blood was collected from the brachial vein into a heparinized capillary and 2–5 μL of blood was blotted onto filter paper (Whatman 1001 125, General Electric, Boston, MA, USA) for subsequent genotyping. The birds were banded with United States Fish and Wildlife Service bands for individual identification before release.

#### Ethics statement

This study was conducted under permits from the U. S. Fish and Wildlife Service (MB08005B-0), the U. S. Department of the Interior (23818), the Virginia Department of Game and Inland Fisheries (053668), and an approved protocol from Virginia Tech’s Institutional Animal Care and Use Committee (protocol 13–074).

### Genotyping

We extracted total genomic DNA from the blood blots using the Qiagen DNeasy Blood and Tissue kit animal blood protocol (Qiagen Inc., Valencia, CA, USA). We modified this protocol for the removal of blood from the filter paper by changing the volumes of Proteinase K and phosphate buffered saline (PBS; 50 mM potassium phosphate, 150 mM NaCl) used per reaction from the volumes of 20 μL kit-supplied Proteinase K and 200 μL PBS recommended in the manufacturer’s protocol to 30 μL and 190 μL, respectively.

We used PCR to amplify 15 microsatellite loci developed for use with Song Sparrows (Table 2). These amplifications were carried out on either a C1000 thermal cycler (Bio-Rad Laboratories, Hercules, CA) or a Veriti™ thermal cycler (Applied Biosystems, Foster City, CA, USA). Each 12.5 μL reaction included 10 mM 10x AmpliTaq Gold buffer, 25.0 μg/ml bovine serum albumin, 2 mM MgCl_2_, 0.15 mM deoxynucleotide triphosphates, 0.5 μM each of fluorescently labeled forward primers and non-labeled reverse primers, 0.5 units of AmpliTaq Gold DNA polymerase (Applied Biosystems, Foster City, CA, USA), and 1 μL DNA template (10–60 ng/μL concentration). These reactions were run with an initial denaturization step of 95°C for 9 min, 40 cycles of 95°C for 30 sec, a locus-specific annealing temperature for 30 sec (Table 2), an extension step at 72°C for 30 sec, with a final elongation step of 72°C for 5 min. We also conducted genetic sex determination using the *P0*, *P2*, and *P8* primers [28] (protocol TF Wright personal communication). PCR products were separated on 2% agarose gels before visualization. Samples showing a single bright fragment were scored as male, and those showing three product fragments were scored as female; females display either two or three fragments, depending on species [28].

**Table 2:** Summary of microsatellite loci.

Locus	Annealing Temperature (° C)	# Genotyped	# Alleles	Allele Size Range	*H*_o_	*H*_e_
*Mme012*	64	198	10	172–234	0.20	0.34
*Sosp001*	64	205	37	219–375	0.88	0.88
*Sosp002*	64	205	6	161–181	0.52	0.51
*Sosp003*	59	194	20	180–252	0.87	0.91
*Sosp004*	60	207	13	175–226	0.75	0.80
*Sosp005*	60	205	8	109–137	0.77	0.76
*Sosp008*	60	205	3	175–183	0.07	0.11
*Sosp010*	64	205	6	140–180	0.49	0.49
*Sosp012*	60	184	23	132–230	0.57	0.89
*Sosp013*	57	204	11	167–215	0.84	0.80
*Sosp014*	60	205	18	214–288	0.60	0.86
*Sosp052*	59	194	16	202–298	0.74	0.83
*Sosp055*	59	172	22	371–460	0.86	0.75
*Sosp062*	59	147	11	344–438	0.64	0.78
*Sosp141*	58	188	8	291–321	0.37	0.60

Microsatellite locus name, annealing temperature (°C), number of individuals genotyped at the locus, number of alleles found at the locus, allele size range (in base pairs), observed and expected heterozygosity. *Mme012* was developed by Jefferey et al. [29]; *Sosp001*, *Sosp002*, *Sosp003*, *Sosp004*, *Sosp005*, *Sosp008*, *Sosp010*, *Sosp012*, *Sosp013*, and *Sosp014* were developed by Sardell et al. [30]; *Sosp052*, *Sosp055*, *Sosp062*, and *Sosp141* were developed by Nietlisbach et al. [31].

Microsatellite PCR products were diluted and combined for fragment analysis with Hi-Di™ Formamide and GeneScan™ 500 LIZ™ dye Size Standard (Applied Biosystems, Foster City, CA, USA). Fragment analysis was performed on an ABI 3130xL Genetic Analyzer at either the University of Texas at El Paso or New Mexico State University; a subset of samples were analyzed on both machines and this analysis confirmed that fragment lengths were comparable across sequencers. Alleles were scored in GeneMapper v.4.0 and reviewed by hand, then binned using FlexiBin v.2 [32].

### Analysis

We used GenePop 4.2 [33,34] to test each locus for conformance to Hardy–Weinberg equilibrium (HWE), both within a sampling site and across all sites, and to determine if there was linkage disequilibrium (LD) between any pairs of loci. We used GenAlEx 6.5 [35,36] to calculate observed and expected heterozygosity for each locus across sites and across loci for each site, and to calculate the inbreeding coefficient *F*_IS_.

We calculated pairwise *F*_ST_ values between each pair of study sites using GenAlEx 6.5 [35,36], using 9,999 permutations to estimate *P*-values for *F*_ST_ between sites. We applied α = 0.05 and a false discovery rate (FDR) [37] of 0.1 when determining the statistical significance of pairwise *F*_ST_ values. We also implemented a Mantel test in GenAlEx to test for the presence of isolation by distance [38].

We conducted a hierarchical AMOVA (analysis of molecular variance) using the R package “poppr” v. 2.8.4 [39]. This analysis partitioned the variation in the molecular data between urbanization categories, between sites within categories, between samples within sites, and within samples (individuals). We used a Monte Carlo randomization test with 999 permutations to assess statistical significance.

We also applied two Bayesian clustering methods to estimate the number of genetic clusters represented by our samples. First, we estimated the number of genetic clusters using Structure 2.3.4 [40], using the admixture model, correlated allele frequencies, and the LOCPRIOR model. Burn-in length was 100,000 replications with 1,000,000 replications after burn-in. We performed this analysis with 10 runs each of *K* from one to 10, then used both the L(K) and Δ(K) methods implemented in Structure [40] and Structure Harvester [41] to determine the most likely number of genetic clusters, as recommended by Janes et al. [42]. After the initial runs to determine the most likely value of K, we conducted an additional Structure run to refine our estimates of cluster membership. We increased the number of replications to 5,000,000, with all other model parameters remaining the same as in earlier analyses.

Analysis in Geneland 4.0.8 [43], which incorporates spatial information about sampling location in its clustering algorithm and has increased power to detect low levels of genetic structure across space, was performed for one to 10 clusters, with coordinate uncertainty of 500 m, 1,000,000 Markov chain Monte Carlo (MCMC) repetitions, a thinning interval of 1,000, the correlated allele frequency model, the spatial model, without considering null alleles, and a secondary burn-in of 500. Coordinate uncertainty was set to 500 m because it was the mean distance between the center of the sampling site and the nest for 57 birds from a separate study for which nest location was known and nest coordinates obtained using a GPS unit (eTrex 20, Garmin, Olathe, KS, USA). After initial Geneland runs to determine the most likely number of genetic clusters, we ran an additional analysis with *K* set at the most likely value to refine estimates of the proportional membership to each cluster for each individual bird. Parameters for this analysis were the same as the initial Geneland runs, except a single run was conducted with the number of MCMC repetitions increased to 5,000,000, and *K* was set at the most likely value obtained from the initial analysis.

## Results

A total of 208 individuals, 94% of which were males, were successfully genotyped (Table 2). We did not detect consistent patterns of departure from HWE for most loci when we tested each locus individually at each site. However, locus Mme012 was out of HWE at six of seven sampling sites and was removed from further analysis. Three other loci were out of HWE at fewer sampling sites; *Sosp014* and *Sosp141* were each out of HWE at two sites, and *Sosp012* was out of HWE at four sites. We detected evidence of linkage disequilibrium between *Sosp003* and *Sosp012*. Consulting the Song Sparrow linkage map [31] confirmed that *Sosp003* and *Sosp012* were in the same linkage group, and that all other loci used in our study were in different linkage groups. *Sosp012* was removed from further analysis because in addition to the LD, it was also out of HWE at four sites. After the removal of *Mme012* and *Sosp012*, 13/15 loci were retained for subsequent analyses.

Observed heterozygosity was generally similar across the seven study sites and did not appear to be related to urbanization index (Table 1). Pairwise *F*_ST_ ranged from 0.010–0.023 and showed no significant genetic differentiation between any pairs of sites (Table 3). Although five of 21 pairwise comparisons had *P*-values < 0.05, none were statistically significant when a FDR = 0.1 was applied. We did not detect any evidence of isolation by distance when testing for correlations between matrices of geographic and genetic distance (Mantel test, *P* = 0.46).

**Table 3:** Pairwise *F*-statistics for pairs of study sites (below the diagonal) grouped by habitat type. *P*-values are above the diagonal; no pairwise comparisons were statistically significant when a FDR = 0.1 was applied.

Suburban-Radford Campus	Suburban-Virginia Tech Campus	Intermediate-Turfgrass Research Center	Intermediate-Smithfield Plantation	Rural-Heritage Park	Rural-Kentland Farm	Rural-Stream Site	
	0.074	0.074	0.052	0.025	0.027	0.212	Suburban-Radford Campus
0.014		0.500	0.089	0.024	0.366	0.636	Suburban-Virginia Tech Campus
0.023	0.012		0.073	0.246	0.312	0.247	Intermediate-Turfgrass Research Center
0.022	0.013	0.023		0.049	0.197	0.225	Intermediate-Smithfield Plantation
0.021	0.014	0.017	0.019		0.028	0.058	Rural-Heritage Park
0.016	0.007	0.014	0.013	0.014		0.054	Rural-Kentland Farm
0.013	0.006	0.014	0.013	0.013	0.010		Rural-Stream Site

Using hierarchical AMOVA, we determined that most of the variation (88.8%) in our data was within samples, which is consistent with panmixia (Monte Carlo test, *P* < 0.001). There was no support for genetic structuring at the level of urbanization categories (Monte Carlo test, *P* = 0.97).

**Fig 2:**
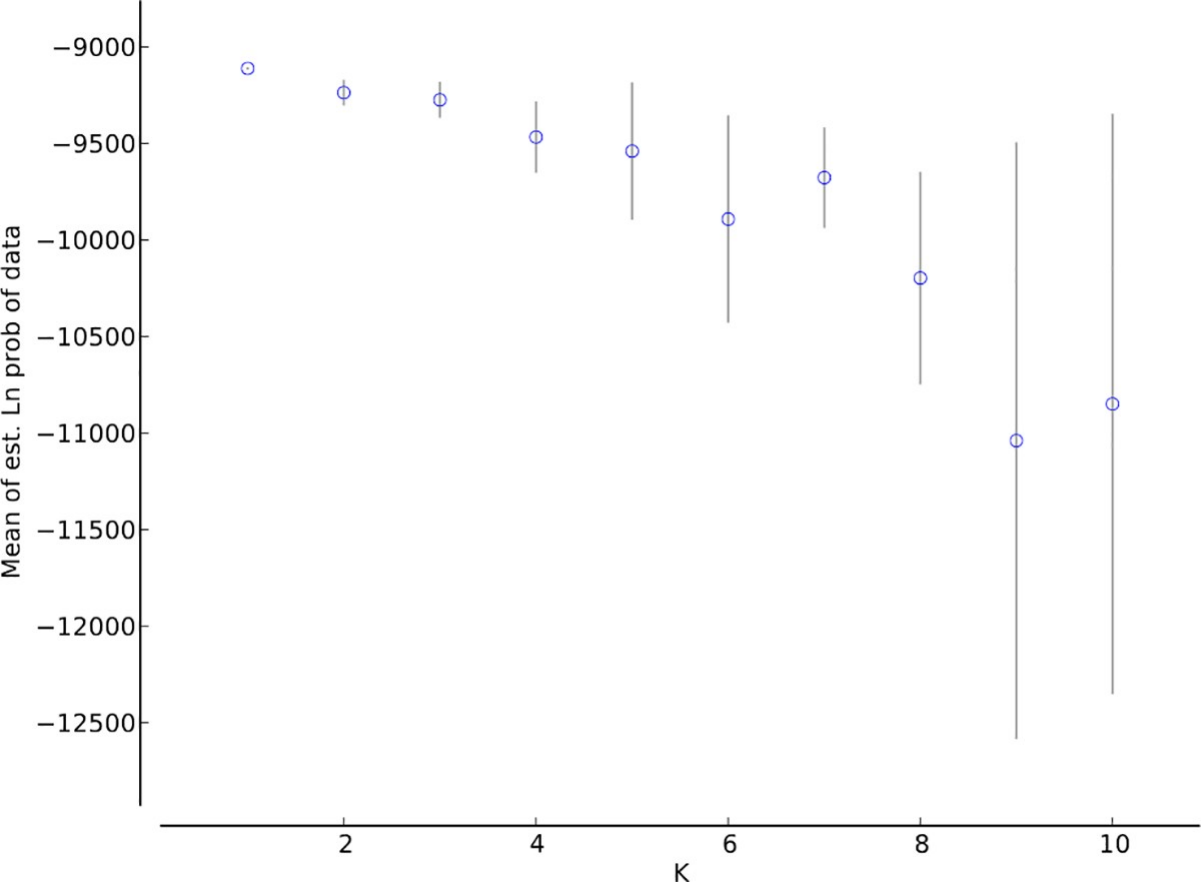
Structure L(*K*) plot, showing that the value of *K* with the highest likelihood is 1.

**Fig 3:**
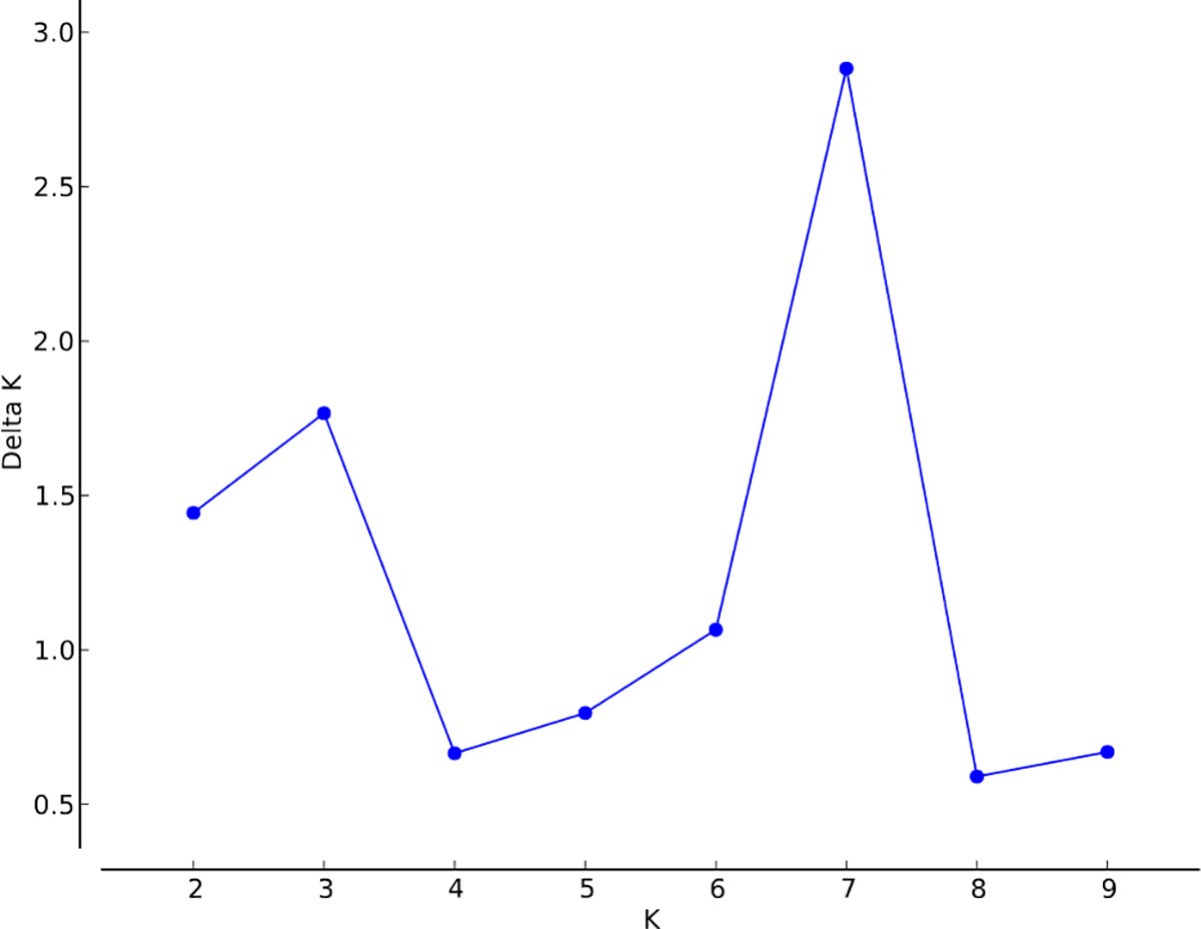
Structure Harvester Δ(*K*) plot, showing peaks for *K* of 3 and 7.

**Fig 4:**
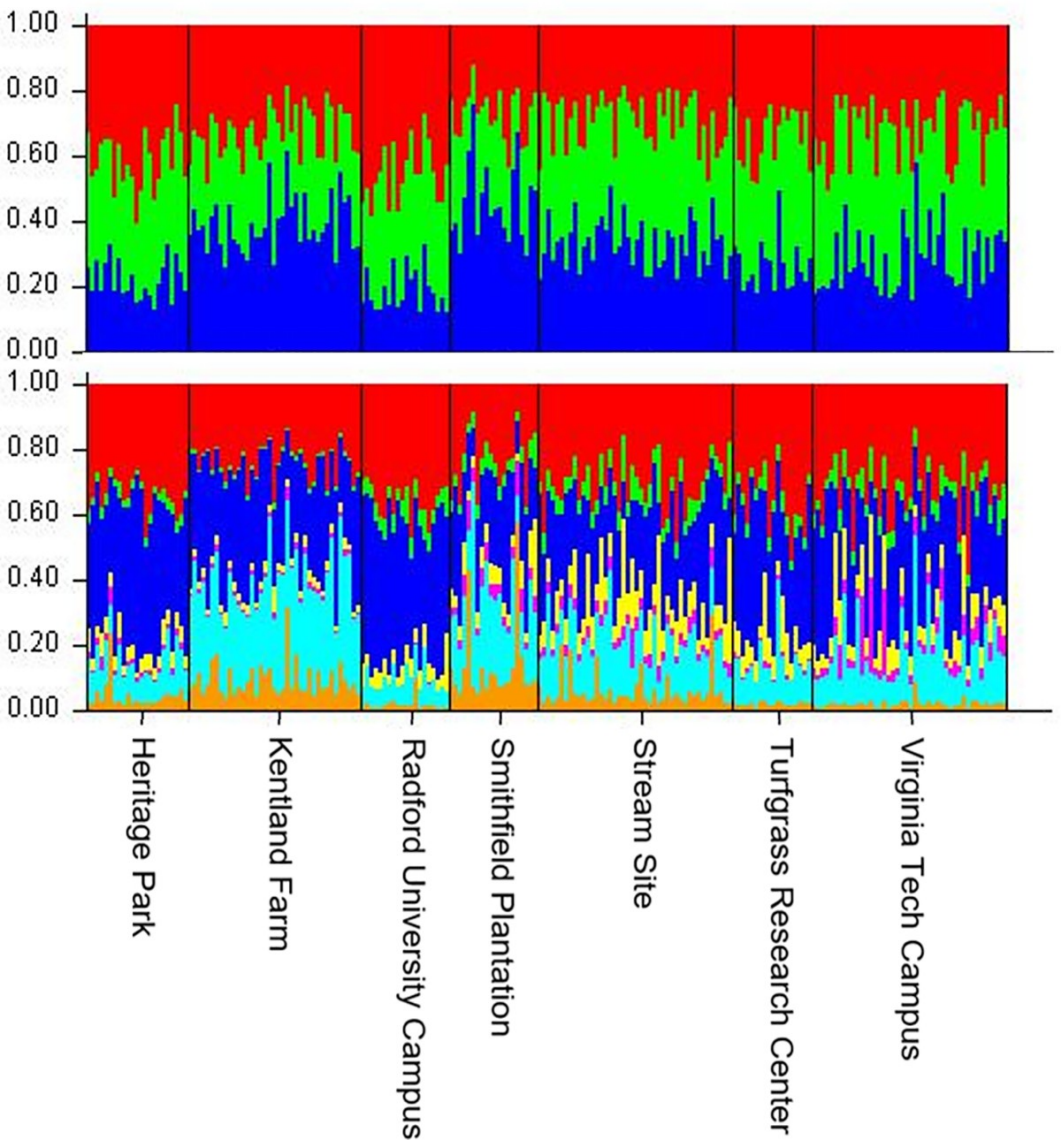
Structure barplot for (a) *K* = 3, (b) *K* = 7, sorted by sampling site and showing proportional membership for each individual in each identified genetic cluster. Each vertical bar represents an individual, each color indicates a cluster, while bar height specifies proportional affiliation to each cluster. The observed pattern, in which all individuals are affiliated similarly with each cluster, is consistent with a lack of population genetic structure.

Using the L(K) method, Structure suggested that the most likely value of *K* was one (Fig 2). We observed the highest Δ(K) value for *K* of seven, with an additional peak at *K* of three (Fig 3). However, the associated Structure barplots showed fairly consistent proportional membership across all purported clusters for all individuals (Fig 4). If *K* were truly greater than one, we would instead expect to see most individuals with predominant membership in a single cluster, with different individuals assigned to different clusters. We interpret these results as evidence that all individuals genotyped were likely to be members of the same genetic cluster; it is impossible to directly test for *K* of one using the Δ(K) method [42], and both L(*K*) and the barplots suggested that that true number of clusters was one.

The initial Geneland analysis identified a most likely *K* of eight, with the two runs with the highest likelihood settling on *K* of eight. Four of ten runs overall indicated that the most likely value of *K* was seven. We ran a follow-up analysis in Geneland with *K* set to eight, in case Geneland analysis had detected cryptic subdivision in one of our seven sampling sites. However, the parameterization run instead found that birds from 6/7 sites were predominantly assigned to the same cluster, and that birds from the seventh site (Kentland Farm) were largely assigned to that cluster as well (Table 4). Mean proportional membership to cluster 6 was ≥ 0.68 for six sampling sites, and > 0.30 for the other two sites (Kentland Farm and Smithfield Plantation), which also had substantial proportional members in cluster 7 (Table 4). Notably, almost no birds were assigned proportional membership to clusters 3 and 5 (Table 4), suggesting that these are “ghost clusters” that are not biologically meaningful. Taken together, these results are largely consistent with a lack of association between either urbanization level or distance and genetic differentiation.

**Table 4:** Mean proportional membership of birds sampled at seven sites to eight genetic clusters identified by Geneland analysis.

Site name	cluster 1	cluster 2	cluster 3	cluster 4	cluster 5	cluster 6	cluster 7	cluster 8
Radford Campus	0.06	0.05	0.00	0.01	0.00	**0.76**	0.09	0.03
Virginia Tech	0.04	0.03	0.01	0.01	0.00	**0.69**	**0.17**	0.05
Turfgrass Research Center	0.04	0.02	0.00	0.01	0.00	**0.68**	**0.16**	0.09
Smithfield Plantation	**0.12**	**0.10**	0.00	0.08	0.00	**0.31**	**0.21**	**0.17**
Heritage Park	0.03	0.01	0.00	0.01	0.00	**0.77**	**0.15**	0.03
Kentland Farm	0.06	**0.17**	0.00	0.01	0.00	**0.33**	**0.36**	0.07
Stream Site	0.04	0.02	0.00	0.02	0.00	**0.73**	**0.15**	0.04

Proportional membership values > 0.10 are shown in bold. Cells with proportional membership values > 0.20 are shaded, with the darkest shading corresponding to the highest values.

## Discussion

We did not find evidence of an effect of low-density urbanization on genetic diversity or genetic structure in Song Sparrows living along a gradient of urbanization in and around Blacksburg, VA. Measures of genetic diversity did not indicate an effect of urbanization; observed and expected heterozygosity were comparable across all sites (Table 2). Further, observed heterozygosity did not appear to be related to urbanization index, nor did we find evidence of either isolation by distance or significant *F*_*ST*_ between sampling sites. Similarly, AMOVA results were most consistent with panmixia, with most molecular variation within individuals rather than between sites or urbanization levels. Finally, results of Bayesian clustering algorithms as implemented in Structure [40] and Geneland [43] were not consistent with differentiated genetic clusters. Overall, our results are consistent with an interpretation that all sites belonged to a panmictic population, suggesting that low-intensity urbanization is not affecting genetic diversity in this population of Song Sparrows, and that the behavioral differences that have been observed between urban and rural birds [25] are instead likely to be a plastic response to environmental heterogeneity.

Why did we fail to detect significant genetic structuring in Song Sparrows across an urbanization gradient, when other studies of the same focal species, using similar numbers of genetic markers, over similar spatial scales, did find such structure (in some cases even in the absence of anthropogenic development) [23,24]? Despite the similarities among studies, one possibility is that our study suffered from insufficient power to detect existing differences, and that using a larger number or different type of molecular markers would have enabled us to detect existing population genetic structure. For example, a recent study in an endangered amphibian used SNPs (single nucleotide polymorphisms) to detect population genetic structure that was not apparent in a related analysis that employed microsatellite loci [44], and the authors determined that 300–400 SNP loci were necessary to detect the structure that existed in their population. Thus, we cannot eliminate the possibility that population genetic differentiation exists in this Song Sparrow population, but at a level that is undetectable using our methods. However, this possibility seems unlikely, given that other studies using similar numbers of loci have detected spatial patterns of genetic structure in Song Sparrows over shorter spatial scales [23,24].

It is also possible that there simply is no genetic differentiation among Song Sparrows living at the sites that we studied. This possibility is consistent with some other studies of population genetic structure in songbirds, which have documented results ranging from little to no genetic differentiation between urban and rural populations, to cases where urban populations unexpectedly had higher genetic diversity than did rural populations. Notably, a previous study of Song Sparrows in the Bay Area of California did not detect significant genetic structuring, even among morphologically recognized subspecies [45]. However, a subsequent study by the same research group and including many of the same samples [23], attributed detected fine-scale genetic structure to a potential lack of gene flow across subspecies boundaries, leading to some difficulty in interpreting the results of the earlier study. Despite the sometimes conflicting results from studies on Song Sparrows, studies of several other songbird species have revealed little to no effect of urbanization on avian genetic structure and diversity. For example, researchers have documented a lack of genetic structure associated with urbanization in House Sparrows (*Passer domesticus*) [14]. In Tree Swallows (*Tachycineta bicolor*), significant genetic structure was not detected, but researchers found higher levels of genetic diversity in less rural areas [46], a result that actually contradicts the usual prediction of decreased genetic diversity with increasing urbanization [45]. Genetic diversity in Golden-cheeked Warblers (*Setophaga chrysoparia*, formerly *Dendroica chrysoparia*) living across a broad expanse of urban area (Central Texas) did not differ between sites, but low, yet significant, levels of genetic differentiation were detected [47]. Similarly, urban and rural Burrowing Owls (*Athene cunicularia*) did not show differences in genome-wide heterozygosity, but the urban populations did significantly display weak genetic structure [48]. Our finding of a lack of an effect of urbanization on genetic structure and diversity is similar to those of other studies that also focused on avian species in areas characterized by low-density anthropogenic fragmentation [14,46]. Such lower intensity urban development may maintain habitat connectivity, thus allowing for more admixture and the retention of more genetic diversity in avian populations. Alternatively, the relative recency of development may explain the lack of effect on population genetic structure in this and other studies.

Finally, although we have focused here on studies of genetic diversity and structure in avian systems, an extensive recent review of the effects of urbanization on both animals and plants suggested that inconsistent and even contradictory results among studies may not be unusual at all [15]. Miles et al. [15] conducted a meta-analysis of 167 studies of gene flow and genetic drift in urban environments published from 1990–2018, most of which started from the same premise as the current study: that urbanization should have negative effects on gene flow and positive effects on genetic structure. Their highly-powered meta-analysis found only a weak signature of urbanization on genetic diversity, and no overarching pattern of genetic differentiation with urbanization. Although the results of many of the studies in their meta-analysis were consistent with reduced gene flow and increased genetic structure with urbanization, many also showed the opposite pattern, or no statistical relationship at all. Thus, Miles et al. [15] end by suggesting that identifying general patterns in the effects of urbanization on population genetics will be difficult, that researchers should also take into consideration factors specific to their study species or landscape that could also influence genetic patterns, and that researchers continue to document patterns of genetic structure across a range of urban landscapes. Despite variation among studies, the pattern appears to be that high intensity anthropogenic development over long periods of time does affect population genetic measures. However, assessing the relative magnitude of these effects will require conducting studies in diverse species and across a range of urbanization intensities, including less-intensely urbanized areas such as those in the current study.

## Supporting information

S1 Data (XLSX)
